# Micromechanical and Tribological Performance of Laser-Cladded Equiatomic FeNiCr Coatings Reinforced with TiC and NbC Particles

**DOI:** 10.3390/ma17194686

**Published:** 2024-09-24

**Authors:** Artem Okulov, Olga Iusupova, Kun Liu, Jie Li, Alexander Stepchenkov, Vladimir Zavalishin, Yulia Korkh, Tatyana Kuznetsova, Krishna Kishore Mugada, Arivarasu Moganraj

**Affiliations:** 1 M.N. Mikheev Institute of Metal Physics, Ural Branch of the Russian Academy of Sciences, Ekaterinburg 620108, Russia; yusupovaolga1@yandex.ru (O.I.); alexander.stepchenkov@gmail.com (A.S.); zavali@imp.uran.ru (V.Z.); korkh@imp.uran.ru (Y.K.); kuznetsova@imp.uran.ru (T.K.); 2 School of Materials Science and Engineering, Jiangsu University of Science and Technology, Zhenjiang 212100, China; liu_kun@163.com (K.L.); lijie081790@163.com (J.L.); 3 Institute of Physics and Technology, Ural Federal University, Ekaterinburg 620002, Russia; 4 Department of Mechanical Engineering, Sardar Vallabhbhai National Institute of Technology, Surat 395007, India; kkm@med.svnit.ac.in; 5 Centre for Innovative Manufacturing Research, Vellore Institute of Technology, Vellore 632014, India; arivarasu.m@vit.ac.in

**Keywords:** short-pulsed laser cladding, equiatomic FeNiCr coating, TiC, NbC, Raman spectroscopy, micromechanical characterization, tribological analysis

## Abstract

This paper discusses a comparative micromechanical and tribological analysis of laser-cladded equiatomic FeNiCr coatings reinforced with TiC and NbC particles. Two types of coatings, FeNiCr-TiC (3 wt.% TiC) and FeNiCr-NbC (3 wt.% NbC), were deposited onto an AISI 1040 steel substrate by means of short-pulsed laser cladding. The chemical composition, microstructure, and micromechanical and tribological characteristics of the coatings were systematically investigated via optical and scanning electron microscopy, Raman spectroscopy, and mechanical and tribological tests. The average thicknesses and compositional transition zones of the coatings were 600 ± 20 μm and 150 ± 20 μm, respectively. Raman spectroscopy revealed that both coatings are primarily composed of a single FCC γ-phase (γ-FeNiCr). The FeNiCr + 3 wt.% TiC coating exhibited an additional TiC phase dispersed within the γ-FeNiCr matrix. In contrast, the FeNiCr + 3 wt.% NbC coating displayed a more homogeneous distribution of finely dispersed NbC phase throughout the composite, leading to enhanced mechanical behavior. Micromechanical characterization showed that the FeNiCr + 3 wt.% NbC coating possessed higher average microhardness (3.8 GPa) and elastic modulus (180 GPa) compared to the FeNiCr + 3 wt.% TiC coating, which had values of ~3.2 GPa and ~156 GPa, respectively. Both coatings significantly exceeded the AISI 1040 steel substrate in tribological performance. The FeNiCr + 3 wt.% TiC and FeNiCr + 3 wt.% NbC coatings exhibited substantial reductions in both weight loss (37% and 41%, respectively) and wear rate (33% and 42%, respectively) compared to the substrate material. These findings indicate that more finely dispersed NbC particles are better suited for hardening laser-cladded equiatomic FeNiCr-NbC coatings, making them advanced candidates for industrial applications.

## 1. Introduction

The development of advanced metal matrix composite (MMC) coatings remains a significant scientific and technological challenge [1,2]. This is driven by the urgent need to enhance the service life of industrial machinery and equipment in today’s economic environment. Among the most commonly known and commercially used materials for high-strength coatings are FeNiCr-based austenitic stainless steels (ASSs) [3]. These materials find application in various industrial sectors: automotive and marine industries, petroleum refining, water supply systems, pollution control, and transportation equipment [4]. Furthermore, such ASS materials are widely employed as in-core components for nuclear light water reactors, as well as turbine discs and gas compressors [5,6,7,8]. It is obvious that the mentioned FeNiCr-based ASSs mainly consist of iron (Fe), while nickel (Ni) and chromium (Cr) serve as effective additives, providing specific structural, phase, and mechanical properties [3]. Nickel enhances the impact toughness, ductility, and strength at various temperatures, significantly improving resistance to corrosion and oxidation. In turn, chromium greatly increases hardenability and primarily improves corrosion resistance in oxidizing environments. The unique chemical composition of ASSs results in exceptional properties such as excellent formability, ductility, weldability, non-magnetism, and superior impact toughness at cryogenic temperatures [9]. The predominant crystal structure of ASSs is face-centered cubic (FCC), which influences their physical, thermal, and mechanical behavior [3,10].

A promising approach to improve the mechanical and tribological performance of conventional ASS coatings is to strengthen them with various carbides. Incorporating ceramics into ASSs promotes the formation of new reinforcing phases, leading to enhancements in their structure and mechanical characteristics [11,12]. The FeNiCr alloy system, especially its equiatomic modification, has been extensively studied [13,14,15,16,17,18,19,20,21]. As reported in [21], the mechanical properties, such as microhardness and elastic modulus, of equiatomic FeNiCr coatings exceed those of AISI 1040 steel, demonstrating its potential for industrial applications. Despite the attractive performance characteristics of these equiatomic materials, there is limited research on the use of reinforcing additives, such as ceramics, to improve their properties [21,22]. Specifically, the same study [21] revealed that a minor addition of 3 wt.% boron carbide (B_4_C) led to a notable enhancement in the microhardness and elastic modulus parameters by 60% and 11%, respectively, compared to the additive-free equiatomic FeNiCr coating.

Along with B_4_C, the addition of titanium (TiC) and niobium (NbC) carbides as reinforcing materials to obtain advanced MMC coatings is of particular interest. As is known, TiC is a refractory ceramic material with a melting point of 3250 °C and a high hardness of 2900–3200 HV_50_, but its low density of 4.92 g/cm^3^ can lead to floating and uneven distribution of the reinforcing phase when introduced into the metal matrix of the coating [23]. In comparison, niobium carbide (NbC) has a higher density of 7.82 g/cm^3^, sufficient thermodynamic stability, and a high hardness of 3200 HV_50_, surpassing titanium carbide in these aspects [23,24,25]. Since the addition of 3 wt.% B_4_C resulted in excellent mechanical properties while maintaining a fairly defect-free structure [21], the same amount of titanium and niobium carbides was used in this study.

The ultimate structure and mechanical behavior of MMC coatings depend not only on the addition of ceramic reinforcements but also on deposition technologies [26]. Electroplating [27], thermal spraying [28], and laser cladding [29,30,31,32,33,34,35] are some of the most widely known coating technologies. Although electroplating is well established and has low processing costs, it has drawbacks such as limited coating thickness and weak interfacial metallurgical bonding [36]. Thermal spraying has higher efficiency, but the coating–substrate interfacial bonding suffers from numerous defects, such as pores and microcracks, leading to early failures and reduced service life [37]. In turn, laser cladding technology offers several advantages, including a low dilution rate, a small heat-affected zone, and reliable metallurgical bonding between the coating and substrate [28]. Nevertheless, it is crucial to consider the factors influencing the formation of various defects, particularly solidification cracking, inherent to the laser cladding process [38,39].

The study builds upon the existing research on laser-cladded FeNiCr-based coatings by focusing on the synergistic effect of TiC and NbC reinforcement. By systematically comparing their impact on microstructure, chemical and phase composition, mechanical properties, and tribological characteristics, this research offers a comprehensive understanding of the advantages and disadvantages of each carbide and enables informed selection for specific applications. This knowledge is crucial for optimizing coating design and extending the service life of industrial machinery and equipment.

## 2. Experiment

### 2.1. Feedstock Powders

The FeNiCr-TiC and FeNiCr-NbC compounds were synthesized by mixing a custom spherical equiatomic FeNiCr powder (Table 1, PJSC “Ashinsky Metallurgical Plant”, Asha, Russia) with 3 wt.% of single-sourced (LLC IPK “Umex”, Ufa, Russia) high-purity (98.5–99%) commercial irregular-shaped TiC and NbC powders, respectively.

The particle size ranges of the FeNiCr, TiC, and NbC powders were 50–150 µm, 1–15 µm, and 1–30 µm, respectively. To ensure a homogenous powder mixture, a standard dry vibratory mixing process was employed using experimental laboratory equipment (UdSU, Izhevsk, Russia). Two corresponding FeNiCr and 3 wt.% TiC or NbC powders were poured into a special mortar and subjected to 10 min of continuous vibration at a frequency of 50 Hz and an amplitude of 5 mm. These specific parameters were carefully chosen to balance thorough mixing with minimal particle damage, resulting in a uniformly distributed powder mixture.

### 2.2. Laser Cladding Parameters

Short-pulsed laser cladding of the FeNiCr + 3 wt.% TiC and FeNiCr + 3 wt.% NbC coatings was carried out onto an AISI 1040 steel substrate (Table 1, LCC “ANEP-Metal”, Ekaterinburg, Russia) with a roughness (R_a_) of ~1 µm. The roughness was measured using a profilometer model 250 (JSC “Caliber”, Moscow, Russia). To form the coatings, experimental laser equipment adapted with a ytterbium fiber laser with a maximum average power of 50 W and a wavelength of 1.065 µm was utilized. Laser cladding was performed by pulses of 40 ns duration in a chamber with a controlled Ar atmosphere. Six samples, three for each coating type, were prepared. All samples measured 7 × 7 × 7 mm. Each sample was dedicated to a specific characterization technique: microstructural (chemical composition), micromechanical (microhardness and elastic modulus), and tribological (weight loss and wear rate).

### 2.3. Optical and Scanning Electron Microscopy

The microstructure and chemical composition of the FeNiCr + 3 wt.% TiC and FeNiCr + 3 wt.% NbC samples were investigated using a light metallographic microscope NEOPHOT-32 equipped with a digital camera (Carl Zeiss AG, Jena, Germany) and scanning electron microscope (SEM) Quanta 200 Pegasus (FEI Company, Eindhoven, The Netherlands) coupled with energy-dispersive X-ray spectroscopy (EDS).

### 2.4. X-ray Diffraction Analysis

The phase composition of the FeNiCr, TiC, and NbC powders was confirmed using a Shimadzu XRD-7000 diffractometer (Shimadzu Corporation, Tokyo, Japan) coupled with a graphite monochromator using CuK_α_ radiation. The diffraction patterns were collected over the 2θ range of 30–120° with a step size of 0.03° and a dwell time of 2 s per step. The X-ray reflections were identified using X’Pert HighScorePlus 3.0.5 software (Malvern Panalytical, Malvern, UK).

### 2.5. Raman Spectroscopy

Raman spectroscopy was used as a highly sensitive method to determine the phase composition of near-surface layers, in particular, the constituent chemical elements of laser-cladded FeNiCr + 3 wt.% TiC and FeNiCr + 3 wt.% NbC coatings. The Raman spectra were measured using the Raman confocal microscope Confotec^®^ MR200 (SOL Instruments, Augsburg, Germany) with a laser excitation wavelength of 532 nm. The laser power directed toward the sample surfaces was 76 mW. The acquisition time was 20 s with 5–10 accumulations per spectral segment. The spectra were recorded using 1200 lines/mm diffraction grating and an electrically cooled charge-coupled detector.

### 2.6. Micromechanical Characterization

The mechanical properties of the resulting FeNiCr + 3 wt.% TiC and FeNiCr + 3 wt.% NbC coatings were evaluated using a NanoTest-600 system (Micro Materials Ltd., Wrexham, UK) equipped with a Berkovich indenter. Microindentation tests (ISO 14577-1:2015 [40]) were performed with a loading/holding/unloading time of 20 s each and a maximum load of 250 mN. During microindentation, 10 impressions were obtained at each depth of both samples (a total of 200 for each coating). The measurement error was determined using standard deviation with a confidence probability of 0.95. The previously reported additive-free equiatomic FeNiCr coating [21] served as a reference material.

### 2.7. Tribological Tests

Tribological tests (analog of ASTM G132-96 [41]) were carried out on experimental laboratory equipment based on a modified shaping machine (IMP UB RAS, Ekaterinburg, Russia). For this, both synthesized FeNiCr-TiC and FeNiCr-NbC coatings, each with a working surface of 7 × 7 mm^2^, were subjected to reciprocating movement against a fixed abrasive—Al_2_O_3_ corundum (LLC “Abraziv”, Chelyabinsk, Russia) with a hardness of ~2000 HV and a grain size of ~160 μm. Each sample was tested twice to obtain statistically relevant data. The test parameters were as follows: normal load, N = 15 N; average sliding speed, V = 0.175 m/s; friction path length, L = 17.6 m; stroke length, l = 100 mm; lateral displacement of the abrasive cloth during one double stroke of 1.2 mm. Wear resistance was evaluated by measuring the weight loss of the tested samples using A&D GR-202 analytical balances (A&D Company Ltd., Tokyo, Japan) with an accuracy of 0.01 mg. The wear rate of each coating was calculated using the following formula [42]:

(1) $$I_{h}=\frac{Q}{\rho \mathrm{SL}}$$

where Q—weight loss, g; ρ—material density, g/cm^3^; S—contact area, cm^2^; L—friction path, cm.

Before testing, all coatings were ground on Al_2_O_3_ abrasive sandpaper to create a flat friction surface with roughness (R_a_) of ~3 µm measured using the profilometer model 250.

## 3. Results and Discussion

### 3.1. Microstructure and Phase and Chemical Composition

**Figure 1:**
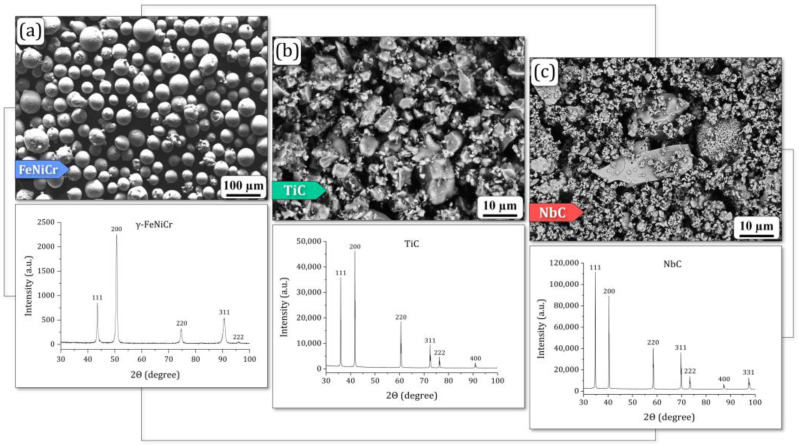
Morphological and XRD characterization of the feedstock powders: (**a**) custom spherical FeNiCr and commercial irregular-shaped (**b**) TiC and (**c**) NbC powders.

The custom spherical FeNiCr powder with commercial irregular-shaped TiC and NbC powders used for the laser cladding process are shown in Figure 1. The fractional, phase, and chemical compositions of the above powders, declared by the manufacturers, were confirmed using XRD and SEM analyses [21,25].

**Figure 2:**
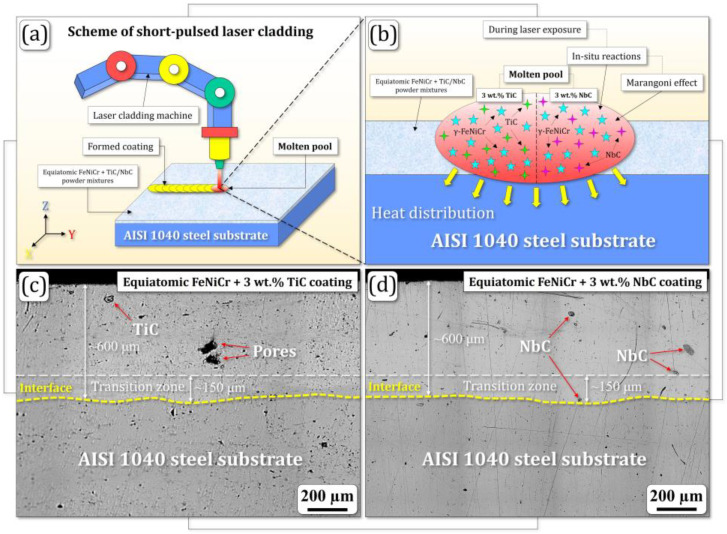
(**a**) Schematic illustration of the laser cladding process accompanied by (**b**) visualization of phase formation and optical cross-sectional images of the (**c**) FeNiCr + 3 wt.% TiC and (**d**) FeNiCr + 3 wt.% NbC samples.

A schematic illustration of the laser cladding process with optical cross-sectional images of the FeNiCr + 3 wt.% TiC and FeNiCr + 3 wt.% NbC samples are shown in Figure 2. In particular, Figure 2b additionally visualizes their phase formation during the laser cladding process (according to the SEM microscopy and Raman spectroscopy data).

Microstructural characterization of both FeNiCr + 3 wt.% TiC and FeNiCr + 3 wt.% NbC coatings revealed uniformly deposited layers with a homogeneous surface texture after HNO_3_ etching. The coatings exhibited a small number of pores and inclusions, consisting of TiC and NbC phases. The coating-substrate interfaces are clearly visible in the compositional transition zones of both samples (Figure 2c,d). The average thickness of the coatings was 600 ± 20 μm.

**Figure 3:**
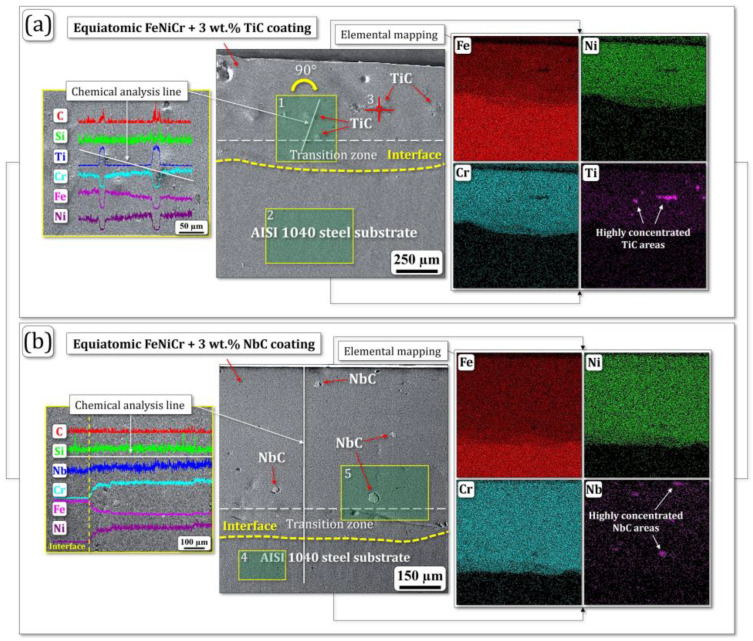
SEM images of the (**a**) FeNiCr + 3 wt.% TiC and (**b**) FeNiCr + 3 wt.% NbC samples. The left and right insets show chemical analysis lines and elemental mappings, respectively.

Elemental mapping of the FeNiCr-TiC and FeNiCr-NbC cross-sections clearly demonstrated compositional transition zones (marked between white and yellow dashed lines; central and right insets in Figure 3a,b). Here, the yellow dashed lines identify the coating–substrate interfaces. According to the right insets (Figure 3a,b), both FeNiCr + 3 wt.% TiC and FeNiCr + 3 wt.% NbC coatings contain small and highly concentrated TiC and NbC areas (clusters), respectively. In particular, the chemical analysis line, depicted in the left inset of Figure 3a, provides compelling evidence for the presence of TiC phases within the FeNiCr-TiC coating. The presence of non-melted TiC and NbC powder particles is likely due to the rapid solidification inherent to short-pulsed laser cladding. The SEM analysis also confirmed that the average thickness of the coatings was 600 ± 20 μm, while their composite transition zones were 150 ± 20 μm.

The transition zones formed during short-pulsed laser cladding of both FeNiCr-TiC and FeNiCr-NbC coatings undergo a series of physical and chemical processes, leading to modifications in the material’s structure and properties [32]. The rapid heating caused by the laser melts the substrate material and partially melts the deposited coating, followed by rapid cooling of the molten pool. This process results in a fine-grained structure within the transition zone, distinct from the coarser grain structure of the substrate. This fine-grained structure imparts higher strength and microhardness to the transition zone compared to the substrate; however, its ductility may be reduced due to the high density of defects formed during rapid cooling.

Simultaneously, diffusion of atoms from the coating and substrate occurs, resulting in the formation of new phases and changes in the chemical composition of the transition zone [21,34]. This diffusion, along with the inherent dilution of the reinforcing carbide additives (TiC or NbC) in the transition zone, contributes to a lower concentration of these carbides compared to the coating itself. This explains the decrease in microhardness values observed in the transition zones compared to the coatings.

The chemical composition of selected areas within the coatings, labeled 1 through 5 in Table 2, provides a detailed analysis of the elemental distribution. The chemical analysis of the FeNiCr + 3 wt.% TiC and FeNiCr + 3 wt.% NbC coatings exhibited a strong correlation with the elemental composition of the feedstock powders, indicating a significant degree of compositional inheritance during the coating deposition process.

In general, this observation highlights that the chosen technological parameters (laser power and scanning speed) of the short-pulsed laser cladding led to the formation of uniform layered coatings with a fairly homogeneous elemental composition.

### 3.2. Raman Spectroscopy Phase Analysis

**Figure 4:**
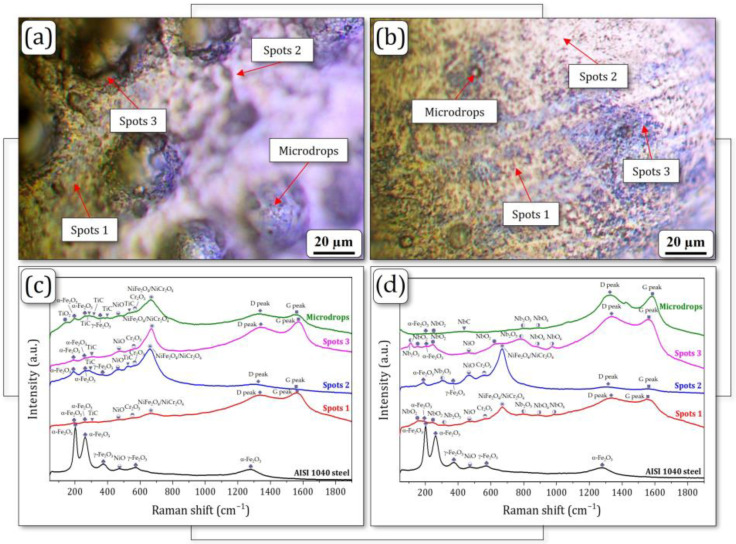
Optical images of spots 1 (grey areas), spots 2 (white areas), spots 3 (dark areas), and microdrops with Raman spectra of the present (**a**,**c**) FeNiCr + 3 wt.% TiC and (**b**,**d**) FeNiCr + 3 wt.% NbC coatings.

Optical images of the characteristic surface areas named spots 1 (grey areas), spots 2 (white areas), spots 3 (dark areas), and microdrops selected on the FeNiCr + 3 wt.% TiC and FeNiCr + 3 wt.% NbC coatings with their corresponding Raman spectra are shown in Figure 4. The Raman peaks, averaged over 5–7 spectra, were identified at each detection area for both samples. The interpretation of the Raman spectra is presented in Table 3.

Raman spectroscopy revealed significant differences in the spectra between the FeNiCr coatings with 3 wt.% TiC or NbC additions and the AISI 1040 steel substrate (Table 3). In contrast with the latter, both coatings exhibited intense amorphous carbon (D and G) peaks, confirming the presence of TiC and NbC phases. The FeNiCr + 3 wt.% TiC coating showed evidence of NiO, Cr_2_O_3_, and NiFe_2_O_4_/NiCr_2_O_4_ phases across all analyzed areas (spots 1, 2, and 3 and microdrops). Similarly, the FeNiCr + 3 wt.% NbC coating contained NiO phase in spots 1, 2, and 3; Cr_2_O_3_ and NiFe_2_O_4_/NiCr_2_O_4_ phases in spots 1 and 2. Importantly, the Cr_2_O_3_ and NiFe_2_O_4_/NiCr_2_O_4_ phases were not detected for the AISI 1040 steel substrate.

The peaks corresponding to the TiC phase [43,44] were also identified for all surface areas (most for the microdrops) of the FeNiCr + 3 wt.% TiC coating (Table 3). At the same time, for different detection areas, the intensity of these peaks was different, which may indicate non-uniform TiC distribution within the coating. In addition, the peak corresponding to the TiO_2_ phase was also detected only for the microdrops, where the most TiC peaks were found. Spots 2 and microdrops of the FeNiCr + 3 wt.% TiC coating are characterized by a high content of carbides, while spots 1 and 3 display only a single peak of titanium carbide.

The peaks corresponding to NbO_2_, Nb_2_O_5_, and NbO_6_ phases, which is associated with the oxidation of Nb atoms in air, were detected for the FeNiCr + 3 wt.% NbC coating (Table 3). At the same time, detection of the NbC phase is difficult [45], and its presence in the coating may appear in the form of detectable niobium oxides [46] and amorphous carbon (D and G) peaks in Raman spectra. The largest amount of niobium oxides, associated with the NbC phase, corresponded to spots 1 and 3. This may indicate a high concentration of NbC particles in these areas. The microdrops were also characterized by high content of niobium carbides in the form of niobium oxides (as previously discussed) and a single NbC peak, whereas spots 2 contained only a single Nb_2_O_5_ peak and low-intensity amorphous carbon peaks.

Overall, all analyzed surface areas of both FeNiCr + 3 wt.% TiC and FeNiCr + 3 wt.% NbC coatings displayed weak α-Fe_2_O_3_, NiO, and Cr_2_O_3_ peaks, as well as intense NiFe_2_O_4_/NiCr_2_O_4_ and amorphous carbon (D and G) peaks, confirming the presence of TiC and NbC phases inside the coatings. Thus, based on the results of a previous study [21] and the Raman spectroscopic analysis presented herein, it can be concluded that both coatings are primarily composed of a single FCC γ-phase (γ-FeNiCr). The FeNiCr + 3 wt.% TiC coating exhibited an additional TiC phase dispersed within the γ-FeNiCr matrix. In contrast, the FeNiCr + 3 wt.% NbC coating displayed a more homogeneous distribution of finely dispersed NbC phase throughout the composite, leading to enhanced mechanical behavior.

### 3.3. Mechanical Characterization

**Figure 5:**
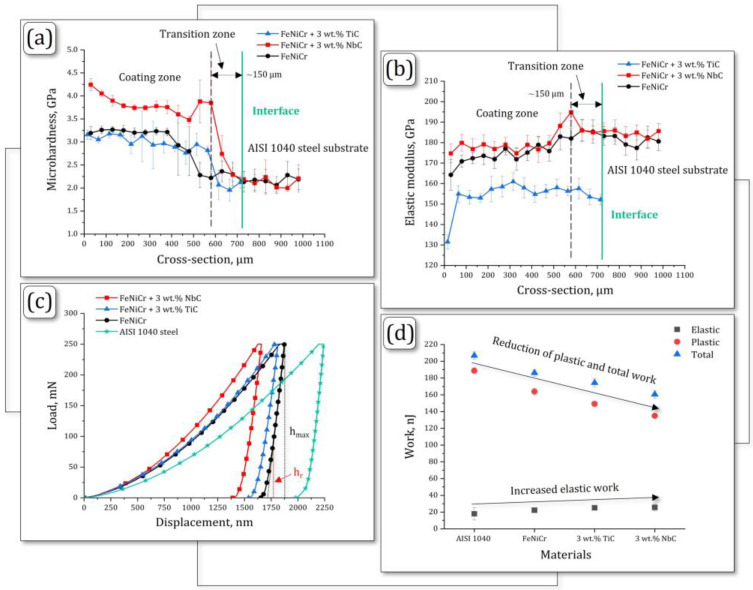
Mechanical characterization of the FeNiCr + 3 wt.% TiC, FeNiCr + 3 wt.% NbC, and additive-free FeNiCr coatings and AISI 1040 steel substrate: (**a**) microhardness, (**b**) elastic modulus, (**c**) loading/unloading curves, and (**d**) work (elastic, plastic, and total) graphs.

According to Figure 5 and Table 4, the average microhardness values (H_IT_) of the FeNiCr + 3 wt.% TiC and FeNiCr + 3 wt.% NbC coatings were ~3.2 GPa and ~3.8 GPa, respectively. This indicated that reinforcement with 3 wt.% NbC led to a noticeable increase in microhardness by ~18% compared to both FeNiCr + 3 wt.% TiC and additive-free FeNiCr (~3.2 GPa) coatings. Additionally, the FeNiCr + 3 wt.% NbC coating microhardness is ~77% higher compared to the AISI 1040 steel substrate (~2.1 GPa). The FeNiCr + 3 wt.% NbC coating exhibits enhanced resistance to both permanent (plastic) and temporary (elastic) deformation, as indicated by its higher H_IT_ value [47]. However, it displays a lower microhardness (~26% less) compared to the previously reported FeNiCr + 3 wt.% B_4_C coating (~5.2 GPa) [21]. This difference in microhardness can be attributed to the presence of harder B_4_C and Fe_2_B phases within the latter.

The microhardness evolution graph (Figure 5a) also visually identified the coating zones without mixing with the AISI 1040 steel substrate material and compositional transition zones, where, on the contrary, they were mixed. The average coating thickness for both samples, confirmed by the microhardness graph, was ~600 µm. The compositional transition zones were ~150 μm. In addition, the above graph (Figure 5a) shows slight deviations from the microhardness values for both coating zones, associated with the redistribution of local TiC and NbC phases in them during the laser cladding process.

The elastic modulus E* (Figure 5b) of the FeNiCr + 3 wt.% NbC coating is significantly higher (by 16% and 5%) compared to the FeNiCr + 3 wt.% TiC and additive-free FeNiCr coatings, respectively. The highest E* value for the FeNiCr + 3 wt.% NbC coating indicates its better resistance to reversible deformation [47,48] compared to the above materials. At the same time, the elastic modulus of the FeNiCr + 3 wt.% NbC coating is almost in accordance (less by ~2%) with the value for the AISI 1040 steel substrate (Table 4) and its value is ~6% less than for the previously reported FeNiCr + 3 wt.% B_4_C coating [21].

The loading/unloading behavior (Figure 5c) demonstrates the effect of reinforcing the equiatomic FeNiCr coating with TiC and NbC particles. The loading/unloading curves were plotted based on the average values of the measurement series range (1–10) for all coatings. When titanium and niobium carbides were added, the maximum indentation depth (h_max_) of both FeNiCr-TiC (1814.98 nm) and FeNiCr-NbC (1660.58 nm) coatings decreased compared to the additive-free FeNiCr coating (1877.43 nm) (Figure 5c). The h_r_ value, specified in Figure 5c, characterizes the point at the intersection of the tangent and unloading curve. The lower h_r_ value indicates better elastic recovery [49,50,51], and its best value (1546.68 nm) corresponded to the FeNiCr + 3 wt% NbC coating (Figure 5c). The elastic, plastic, and total work were calculated using the areas below the loading and unloading curves, respectively, as shown in Figure 5d. As opposed to the AISI 1040 steel substrate material and the additive-free FeNiCr coating, the FeNiCr + 3 wt.% TiC and FeNiCr + 3 wt.% NbC coatings are characterized by a decrease in plastic and total work, and, conversely, an increase in elastic work (Table 4).

Compared to the additive-free FeNiCr coating, the following H_IT_/E∗, H_IT_^3^/E∗^2^, and R_e_ parameters of the FeNiCr + 3 wt.% TiC coating increased by 11%, 27%, and 14%, respectively, while for the FeNiCr + 3 wt.% NbC coating they increased by 13%, 55%, and 16%, respectively. Such a significant increase in the H_IT_/E∗ and H_IT_^3^/E∗^2^ values indicates that the coatings have become stiffer (possess improved resistance to plastic deformation) and more wear-resistant [47,48]. In particular, the observed increase in the R_e_ parameter also specifies the improved ability of the FeNiCr + 3 wt.% TiC and FeNiCr + 3 wt.% NbC coatings to elastically resist mechanical stress up to plastic deformation [50,51,52]. The plasticity δ_A_ index of the FeNiCr + 3 wt.% TiC and FeNiCr + 3 wt.% NbC coatings, on the contrary, decreased by 3% and 5% compared to the additive-free FeNiCr coating, respectively. This confirms the greater hardening of the above coatings (more significant for the FeNiCr + 3 wt.% NbC coating) as a result of reinforcement with TiC and NbC particles.

The lack of a significant microhardness enhancement in the equiatomic FeNiCr coating upon the addition of 3 wt.% TiC can be attributed to a complex interplay of factors: (1) non-uniform powder dispersion (distribution); (2) powder morphology; (3) weak particle-matrix interfacial bonding; and (4) melt pool dynamics. While the presence of TiC and NbC particles was confirmed, the distribution of these particles (more significant for TiC) within the FeNiCr matrix was non-uniform and clustered (as discussed in chapter 3.1), probably due to the rapid solidification inherent to short-pulsed laser cladding. This could limit the effectiveness of TiC as a hardening agent since a more homogeneous distribution is generally required for optimal strengthening effects. The TiC particles were relatively large compared to the NbC particles (Figure 1b,c) and irregularly shaped, which could limit their ability to effectively impede dislocation movement within the FeNiCr matrix. Thus, more finely dispersed NbC particles were found to be better for increasing microhardness (Figure 5a). Similarly, the interfacial bonding between the TiC particles and the FeNiCr matrix may have been weak due to the rapid solidification during laser cladding. This could lead to reduced load-bearing capacity and limited hardening effects, as stress transfer from the FeNiCr matrix to the TiC particles was inefficient. Moreover, the melt pool dynamics during laser cladding could affect the distribution and morphology of TiC particles and thus limit their ability to enhance microhardness.

### 3.4. Tribological Analysis

**Figure 6:**
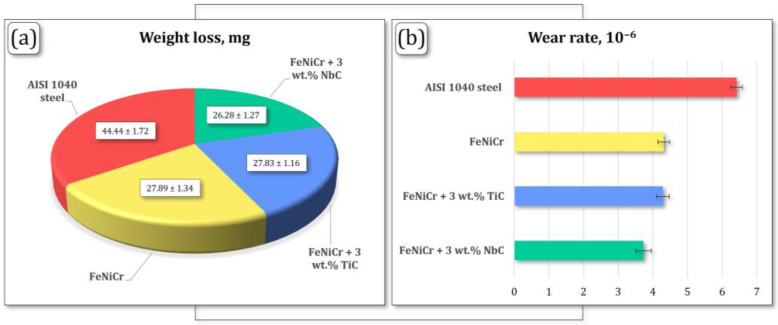
(**a**) Weight loss and (**b**) wear rate values of the FeNiCr + 3 wt.% TiC, FeNiCr + 3 wt.% NbC, and additive-free FeNiCr coatings and AISI 1040 steel substrate.

There were no significant differences in tribological parameters, weight loss, and wear rate between the FeNiCr + 3 wt.% TiC and additive-free FeNiCr coatings (Figure 6 and Table 5).

The weight loss and wear rate parameters for the FeNiCr + 3 wt.% NbC coating were ~6% and ~13% lower, respectively, compared to both FeNiCr + 3 wt.% TiC and additive-free FeNiCr coatings. The most critical differences were revealed between the synthesized FeNiCr + 3 wt.% TiC, FeNiCr + 3 wt.% NbC coatings and the AISI 1040 steel substrate. The weight losses of the FeNiCr-TiC and FeNiCr-NbC coatings were 37% and 41% lower, respectively, compared to the AISI 1040 steel substrate (Figure 6a). In turn, the wear rates for both coatings were also 33% and 42% lower, respectively, compared to the substrate material (Figure 6b).

Thus, micromechanical testing combined with tribological analysis of the synthesized FeNiCr + 3 wt.% TiC and FeNiCr + 3 wt.% NbC coatings demonstrated that reinforcement with more finely dispersed NbC particles is best suited for obtaining harder, defect-free FeNiCr-NbC coatings, making them advanced candidates for industrial applications.

## 4. Conclusions

The study presented in this paper investigates the micromechanical and tribological performance of laser-cladded equiatomic FeNiCr coatings reinforced with TiC and NbC particles. The following main results were obtained:

(1) The optical- and SEM-based microstructural analysis of the coatings demonstrated that their HNO_3_-etched cross-sections possess a uniformly deposited layer and contain a fairly homogeneous surface texture with a small number of pores and inclusions in the form of TiC and NbC phases. Their average thicknesses and compositional transition zones were 600 ± 20 μm and 150 ± 20 μm, respectively.
(2) Raman spectroscopy revealed that both coatings are primarily composed of a single FCC γ-phase (γ-FeNiCr). The FeNiCr + 3 wt.% TiC coating exhibited an additional TiC phase dispersed within the γ-FeNiCr matrix. In contrast, the FeNiCr + 3 wt.% NbC coating displayed a more homogeneous distribution of finely dispersed NbC phase throughout the composite, leading to enhanced mechanical behavior.
(3) Micromechanical characterization showed that the FeNiCr + 3 wt.% NbC coating exhibited higher average microhardness (3.8 GPa) and elastic modulus (180 GPa) compared to the FeNiCr + 3 wt.% TiC coating, which had values of ~3.2 GPa and ~156 GPa, respectively.
(4) Both coatings significantly exceeded the AISI 1040 steel substrate in tribological performance. The FeNiCr + 3 wt.% TiC and FeNiCr + 3 wt.% NbC coatings exhibited substantial reductions in both weight loss (37% and 41%, respectively) and wear rate (33% and 42%, respectively) compared to the substrate material.
(5) Reinforcement with finer NbC particles has a more significant effect on improving the mechanical and tribological performance of laser-cladded equiatomic FeNiCr-NbC coatings, making them advanced candidates for industrial applications.

## Figures and Tables

**Table 1:** The chemical composition of the FeNiCr, TiC, NbC powders, and AISI 1040 steel (wt.%).

Material	Fe	Ti	Nb	Ni	Cr	Mn	C	O	S	P	Si
FeNiCr	Base	-	-	35.6	29.8	-	0.37	-	<0.001	0.008	1.62
AISI 1040 steel		-	-	-	-	0.6–0.9	0.37–0.44	-	≤0.05	≤0.04	0.15–0.35
TiC	-	80.8	-	-	-	-	18.0	1.2	-	-	-
NbC	-	-	89.05	-	-	-	10.3	0.65	-	-	-

**Table 2:** Chemical composition of the FeNiCr + 3 wt.% TiC and FeNiCr + 3 wt.% NbC samples (wt.%).

Selected Area	Fe	Ni	Cr	Ti	Nb	C
1	36.62	29.30	28.75	3.72	-	1.61
2	98.57	0.68	0.41	-	-	0.34
3	4.62	1.28	2.57	83.71	-	7.82
4	98.72	0.59	0.32	-	-	0.37
5	33.30	31.59	30.85	-	2.87	1.39

**Table 3:** Raman spectra detected for different areas of both FeNiCr + 3 wt.% TiC and FeNiCr + 3 wt.% NbC coatings.

**AISI 1040 Steel**	**FeNiCr + 3 wt.% TiC** **Peak Maximum Position, cm^−1^**				**Interpretation**
	**Spot** **s** **1**	**Spot** **s** **2**	**Spot** **s** **3**	**Microdrops**	
-	-	-	-	149	TiO_2_
202	196	191	191	197	α-Fe_2_O_3_
262	260	266	266	264	α-Fe_2_O_3_
-	-	283	-	285	TiC
-	311	-	306	316	TiC
373	-	367	-	374	γ-Fe_2_O_3_
-	-	-	-	398	TiC
470	472	468	475	470	NiO
-	-	526	-	526	TiC
-	547	556	549	560	Cr_2_O_3_
570	-	-	-	-	γ-Fe_2_O_3_
-	665	661	669	665	NiFe_2_O_4_/NiCr_2_O_4_
1276	-	-	-	-	α-Fe_2_O_3_
-	1338	1315	1345	1327	Amorphous carbon (D peak)
-	1561	1548	1572	1555	Amorphous carbon (G peak)
**AISI 1040 Steel**	**FeNiCr + 3 wt.% NbC** **Peak Maximum Position, cm^−1^**				**Interpretation**
	**Spots 1**	**Spots 2**	**Spots 3**	**Microdrops**	
-	-	-	112	-	Nb_2_O_5_
-	163	-	153	-	NbO_2_
202	195	189	197	198	α-Fe_2_O_3_
-	245	-	244	249	NbO_2_
262	-	-	-	-	α-Fe_2_O_3_
-	310	303	-	-	Nb_2_O_5_
373	-	370	-	-	γ-Fe_2_O_3_
-	-	-	-	434	NbC
470	472	466	461	-	NiO
-	560	555	-	-	Cr_2_O_3_
570	-	-	-	-	γ-Fe_2_O_3_
-	-	-	638	-	NbO_2_
-	671	667	-	-	NiFe_2_O_4_/NiCr_2_O_4_
-	797	-	782	799	Nb_2_O_5_
-	887	-	878	890	NbO_6_
-	984	-	973	-	NbO_6_
1276	-	-	-	-	α-Fe_2_O_3_
-	1334	1310	1332	1326	Amorphous carbon (D peak)
-	1549	1560	1560	1583	Amorphous carbon (G peak)

**Table 4:** Mechanical characteristics of the FeNiCr + 3 wt.% TiC, FeNiCr + 3 wt.% NbC, and additive-free FeNiCr coatings and AISI 1040 steel substrate.

Material	Work, nJ			H_IT_, GPa	E*, GPa	H_IT_/E^*^	H_IT_^3^/E*^2^, GPa	R_e_, %	δ_A_
	Elastic	Plastic	Total						
AISI 1040 steel	18.02	188.79	206.81	2.154	184.68	0.0117	0.0003	3.85	0.90
FeNiCr	22.30	163.84	186.14	3.228	172.11	0.0188	0.0011	5.90	0.86
FeNiCr + 3 wt.% TiC (abbreviated as TiC)	25.16	149.12	174.28	3.235	155.71	0.0208	0.0014	6.71	0.83
FeNiCr + 3 wt.% NbC (abbreviated as NbC)	25.68	134.80	160.48	3.810	180.09	0.0212	0.0017	6.86	0.82
**Comparative analysis (change (↓↑) in %)**									
TiC vs. AISI 1040 steel	40 ↑	21 ↓	16 ↓	50 ↑	16 ↓	78 ↑	367 ↑	74 ↑	8 ↓
NbC vs. AISI 1040 steel	43 ↑	29 ↓	22 ↓	77 ↑	2 ↓	81 ↑	467 ↑	78 ↑	9 ↓
TiC vs. FeNiCr	13 ↑	9 ↓	6 ↓	0	10 ↓	11 ↑	27 ↑	14 ↑	3 ↓
NbC vs. FeNiCr	15 ↑	18 ↓	14 ↓	18 ↑	5 ↑	13 ↑	55 ↑	16 ↑	5 ↓
NbC vs. TiC	2 ↑	10 ↓	8 ↓	18 ↑	16 ↑	2 ↑	21 ↑	2 ↑	1 ↓

**Table 5:** Tribological characteristics of the FeNiCr + 3 wt.% TiC, FeNiCr + 3 wt.% NbC, and additive-free FeNiCr coatings and AISI 1040 steel substrate.

Sample	Weight Loss, mg	Wear Rate, I_h_
FeNiCr + 3 wt.% NbC	26.28 ± 1.27	(3.73 ± 0.18) × 10^−6^
FeNiCr + 3 wt.% TiC	27.83 ± 1.16	(4.29 ± 0.18) × 10^−6^
FeNiCr	27.89 ± 1.34	(4.31 ± 0.21) × 10^−6^
AISI 1040 steel	44.44 ± 1.72	(6.42 ± 0.25) × 10^−6^

## Data Availability

Data will be made available upon request.
